# Survey on neutralizing antibodies against Zika virus eighteen months post-outbreak in two southern Thailand communities

**DOI:** 10.1186/s12879-020-05654-8

**Published:** 2020-12-03

**Authors:** Theerut Densathaporn, Rassamee Sangthong, Monvaris Sakolnapa, Smonrapat Surasombatpattana, Marisa Kemapunmanus, Promsin Masrinoul, Sutee Yoksan, Edward B. McNeil, Virasakdi Chongsuvivatwong

**Affiliations:** 1 Epidemiology Unit, Faculty of Medicine, Prince of Songkla University, Hatyai, 90110 Thailand; 2 Immunology and Virology Unit, Department of Pathology, Faculty of Medicine, Prince of Songkla University, Hatyai, 90110 Thailand; 3 Center for Vaccine Development, Institute of Molecular Biosciences, Mahidol University, Nakhon Pathom, 73170 Thailand

**Keywords:** Zika virus, Seroprevalence survey, Cross-protection

## Abstract

**Background:**

In 2016 and 2017, Zika virus (ZIKV) infection outbreaks occurred in two communities in southern Thailand. This re-immerging infection can widely spread by mosquito bites and cause serious complications in a central nervous system among children born to infected mothers. Thus, they should be protected. This study aims to (1) To determine the prevalence of neutralizing ZIKV antibodies in the post-outbreak areas among the general population and pregnancy women residing at various distances from the houses of the nearest index patients; (2) To examine the cross-neutralizing capacity of antibodies against ZIKV on other flaviviruses commonly found in the study areas; (3) To identify factors associated with the presence of neutralizing ZIKV antibodies.

**Methods:**

The two post-outbreak communities were visited at 18 months after the outbreaks. We enrolled (1) 18 confirmed ZIKV infected (index) cases, (2) sample of 554 neighbors in the outbreak areas who lived at various distances from the index patients’ houses, (3) 190 residents of non-outbreak areas, and (4) all pregnant women regardless of gestational age residing in the study areas (*n* = 805). All serum specimens underwent the plaque reduction neutralization test (PRNT). Ten randomly selected ZIKV seropositive and ten randomly selected seronegative specimens were tested for dengue virus serotypes 1–4 (DENV1–4) and Japanese encephalitis virus (JEV) antibodies using PRNT90. Serum titer above 1:10 was considered positive. Multiple logistic regression was used to assess factors associated with seropositivity.

**Results:**

Out of all 18 index cases, 9 remained seropositive. The seroprevalence (95% CI) in the two outbreak areas were 43.7% (35.9–51.6%) and 29.7% (23.3–36.0%) in general population, and 24.3% (20.1–28.8%) and 12.8% (9.7–16.5%) in pregnant women. Multivariate analysis showed that seropositivity was independent of the distance gradient from the index’s houses. However, being elderly was associated with seropositivity. DENV1–4 and JEV neutralizing antibodies were present in most ZIKV-positive and negative subsamples.

**Conclusion:**

Protective herd immunity for ZIKV infection is inadequate, especially among pregnant women in the two post-outbreak areas in southern Thailand.

**Supplementary Information:**

The online version contains supplementary material available at 10.1186/s12879-020-05654-8.

## Background

Zika virus (ZIKV) is a flavivirus that causes acute febrile illness [1–3]. Serious complications include congenital neurological syndrome from vertical transmission and Guillain-Barre syndrome [4–8]. In the last decade, ZIKV epidemics occurred in many Pacific islands, South America, and other countries around the world. Globally, 87 countries in 4 continents have reported ZIKV outbreaks with a total cumulative number of nearly one million cases since 2015 [9–11].

In Southeast Asia, there were evidence of the existence of neutralization antibodies against ZIKV from the serological surveys in the population of the region between 1960 and 1980s [12–14]. Since then, no cases were reported in Thailand until 2013 when two foreigners visiting the country were found to have contracted ZIKV after they returned home [15, 16]. Domestically, 7 confirmed ZIKV infected citizens were reported in Thailand during 2012–2014 [17]. After the rising public awareness of ZIKV from the South American outbreaks in 2015–2016, 1121 confirmed ZIKV infected cases were detected in 43 provinces in 2016 and 577 cases in 33 provinces in 2017 [18]. These alarming figures raised concern on the population at risk for future outbreaks.

Neutralizing antibodies are an important protective element against virus infection. Knowledge of their prevalence against ZIKV can allow epidemiologists to evaluate whether a population has enough immunity to prevent an outbreak. Theoretically, the proportion of immune population greater than 1–1/Basic reproduction number(R0) is required to eliminate the infection by maintaining reproduction number less than 1 [19]. R0 of ZIKV in tropical areas varied from minimum of 1.22 to maximum of 6.9 [20]. Taking the maximum value of 6.9, the prevalence needed to stop the transmission would be up to 85.5%. Such information on the prevalence can assist in the evaluation of the worthiness of developing a ZIKV vaccine for the country.

The cross-reaction of antibodies against different flaviviruses has been well documented. It is, however, not known whether other endemic types of flavivirus in Thailand such as dengue and Japanese encephalitis contribute to the protection of the newly resurgent ZIKV.

In 2016, two ZIKV outbreaks occurred in southern Thailand, one in Surat Thani province during September–November and the other in Narathiwat province, 500 km away from the first outbreak site, during November 2016–January 2017. We took this opportunity to conduct a serological survey to find the answers to the abovementioned knowledge gaps. The objectives of this study were to determine the prevalence of neutralizing ZIKV antibodies among general population and pregnant women in the outbreak areas, examine the cross-neutralizing capacity of ZIKV antibodies against different types of flavivirus infection, and identify factors associated with the presence of neutralizing ZIKV antibodies. We hypothesized that increasing proximity to an index house would increase the likelihood of having neutralizing antibodies against ZIKV.

## Methods

### Study settings

An outbreak district (or District A) in Surat Thani province has a total area of 835.1 km^2^. It is characterized by plain areas surrounded by hills, forests, and rubber plantations. Its population of 50,905 resided in 17,337 households. The other outbreak district (or District B) is located in Narathiwat province, one of the southernmost provinces of Thailand. It has a total area of 372.6 km^2^. Small rivers run from the mountains creating peat swamp forests in the area. The local population of 47,965 resided in 11,285 households [21].

During the outbreaks, local health officers followed the national guideline [22] to control the infection. All index patients underwent reverse transcriptase-polymerase chain reaction (RT-PCR) testing for disease confirmation. The guideline also included the screening of their household contacts, and all pregnant women in the outbreak districts for the infection by the same test. Furthermore, intensive space spraying of insecticide and mosquito surveillance were implemented in the whole affected village area. Our research team retrospectively reviewed the medical records at 1 month after the end of the outbreak and had the meetings with the health officers to plan our current study.

**Fig. 1:**
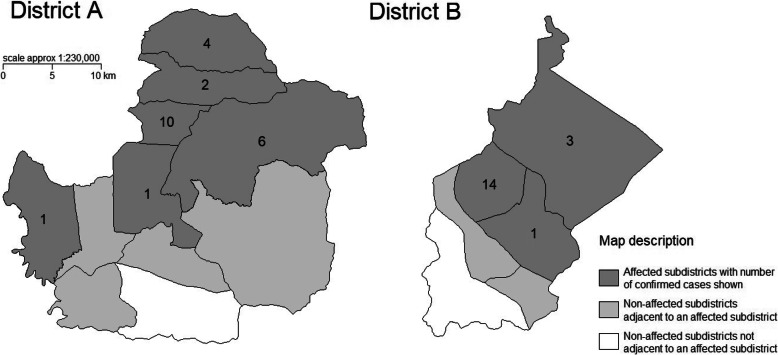
Maps of District A and B showing type of study site and the number of confirmed ZIKV infected cases in each subdistrict. These maps were generated with R software based on the spatial data from GADM website [23, 24]

The review and the meetings revealed that ZIKV infection were confirmed by RT-PCR in 24 and 18 patients in District A and B, respectively. The outbreak covered 12 villages in 6 subdistricts of District A and 5 villages in 3 subdistricts of District B. Figure 1 displays maps of both districts. Dark grey areas denote affected subdistricts with the number of confirmed ZIKV cases. Light grey areas are subdistricts adjacent to the outbreak districts and white areas denote non-adjacent and non-affected subdistricts.

### Study design

A cross-sectional serological survey was conducted in the two affected districts approximately 18 months after each outbreak. In each study district, we recruited two study populations, non-pregnant adults and pregnant women. Details in sampling technique for each group are as follow.

1. *Non-pregnant adults*

Based on the preceding outbreak records, we recruited all index cases, their household contacts, and a random sample of residents who lived varying distances from the nearest index case’s house. All study subjects were at least 18 years old and lived in the area for more than 18 months. Exclusion criteria included pregnancy, immunodeficiency disease, and current use of immunosuppressive drugs.

In order to test our hypothesis, we stratified the non-case population into five groups based on the distance from their household to the house of the nearest index case.

i. Household members of the index cases.
ii. Other residents of the subdistrict where the number of the confirmed cases was highest, who lived within 100 m from an index case
iii. Similar to ii but the distance to the nearest index case was between 101 and 400 m
iv. Similar to ii but the distance to the nearest index case was between 401 and 1000 m
v. Residents of a randomly selected village in a non-affected and non-adjacent subdistrict (white areas shown in Fig. 1)

Based on the limitation of a finite number of cases and the population, for each district, we planned to recruit 400 eligible non-pregnant including all of the index’s household members (i) and addition to a hundred of their neighbors who lived within 100 m (ii), and 100 non-pregnant subjects in each other three distance stratum (iii, iv, and v). /..Having a significance level set to 0.05 and a power of 80%, this sample size would allow us to detect a 20% difference in the prevalence of ZIKV neutralizing antibodies among the distance strata.

2. *Pregnant women*

All pregnant women aged 18 years or above, attending an antenatal care clinic, and living in one of the same districts as a case for more than 12 months were recruited. Exclusion criteria included known cases of ZIKV identified during the outbreak period, major psychiatric or physical illness, cognitive impairment, inability to communicate in Thai, immunocompromised, rheumatologic disorders, and autoimmune diseases. Four hundred pregnant women per district were recruited regardless of their gestational age and distance from their house to the house of the nearest index case. This sample size was calculated based on initial expected seroprevalence of 20%, +/− 4% and alpha = 0.05. They were treated as a separate stratum in the descriptive analysis, but we did not analyze for risk factors among pregnant women because the risk behavior information was not available.

### Data collection

In the non-pregnant adult group, the survey was conducted in District A during March–May 2018 and District B during July–September 2018. A team of local health volunteers were trained as research assistants and instructed to recruit participants and conduct the interviews. The recruitment process involved visiting potential participants at their home, explaining to them the objectives of the study, and requesting their informed consent to participate in the study. Consenting participants were interviewed using a structured questionnaire to collect individual and household information. The distance between the center of each participant’s household and the nearest index case’s household was estimated using Google Maps®. The participants were invited to the health centers for venipuncture at the end of the week where a 10-mL blood sample was taken by a local health officer.

Between July 2018 and May 2019 in District A and B, consecutive pregnant women who attended the antenatal clinics were invited by the research team. Informed consent was obtained. Then a blood sample was taken for serological test. At least 30 min after venipuncture, each blood specimen was centrifuged at 3200 rpm, divided into 4 aliquots and stored at − 20 °C in a freezer at the district hospital, and finally shipped in lots to the Center of Vaccine Development (CVD), Institute of Molecular Biosciences, Mahidol University in Bangkok as it is the WHO-approved reference laboratory on serology and virology for arboviruses.

### Laboratory tests

Plaque reduction neutralization tests (PRNT) for ZIKV-neutralizing antibodies were performed using the following procedures. Rhesus monkey kidney epithelial cells (LLC-MK2) were first seeded in 6-well plates at 1 × 10^5^ cells/well for 7 days. The serum samples were four-fold serially diluted by phosphate buffer solution pH 7.5 with 30% fetal bovine serum, and then mixed with Zika virus strain MR766 at 50 plaque-forming unit (pfu)/well (for a final starting dilution of 1:10) for 1 h. Following infection, cells were overlaid with Dulbecco’s Modified Eagle Medium containing fetal bovine serum, 3.0% carboxymethyl cellulose, and neutral red. Plaques were visualized and counted at 7 days after infection. Probit analysis was used to determine the titer and interpreted as a PRNT50 and later on PRNT90 titer per a reviewer’s suggestion which is the reciprocal of the dilution showing a 50 and 90% reduction, respectively, in plaque count. A neutralization titer ≥1:10 by PRNT90 was considered as a seropositive [25, 26].

Random samples of 10 positive (PRNT90 titers> 1:10) and 10 negative (PRNT90 titer < 1:10) serum samples were used to further test for neutralizing antibody against dengue virus (DENV) serotype 1–4 (strain 16,007,16,681 16,562, and C036/06 respectively) and Japanese encephalitis virus (Beijing strain).

### Statistical analysis

The main outcome variable was whether the subject had a positive neutralizing antibody defined by a PRNT90 titer above 1:10. The detailed titer was further analyzed against the titer of neutralizing antibodies against other types of flavivirus.

The main independent variable was the distance from the participant’s household to the nearest index case’s house. Other independent variables were personal characteristics of the subjects such as age, occupation, behavior related to protective measures against mosquito bites such as the use of mosquito repellents, domestic garbage management, and self-reported history of dengue and chikungunya infection. Prevalence estimation, statistical tests, and the regression in non-pregnant data were computed using the ‘survey’ package to adjust the standard errors based on the sampling weights [27].Variations in seroprevalence among different geographical locations were observed in previous studies [28–30], thus the seroprevalence in the two districts were described separately. For non-pregnant participants, the estimated prevalence of seropositive cases was stratified by the distance band between the household of the participant and the household of the nearest index case. Chi-square test was used to initially determine whether there is a significant difference between seroprevalence of affected and non-affected subdistrict. Proportional trend test was used to determine whether there is a linear trend in the prevalence across gradient of distance from the affected subdistrict.

In order to inquire more power to examine associated factors of seropositivity, the data of the two districts were combined and ‘district’ was handled as one of the independent variables. Predictors for seropositivity from non-pregnant adults were tested using the Rao-Scott Chi-Square test. Independent variables that showed an initial association with ZIKV seropositivity (*P*-value< 0.2) were included in the multivariate logistic regression model to adjust for potential confounding effects. Likelihood ratio test (LR test) is used to test whether the model with that predictive factor is fit the data significantly better than the more restrictive model. Wald test is used to test whether removing of that level is substantially harm the fit of the model.

For cross-neutralization, titers of neutralizing antibodies against ZIKV were plotted against those of the flaviviruses, one-by-one, on a logarithmic scale.

Statistical significance was set at 0.05. All statistical analysis was undertaken using R software.

## Results

The overall response rate of non-pregnant participants was 74.8% (377 out of 504 participants) and 77.2% (385 out of 499 participants) from District A and B, respectively. Half from a total of 18 index cases remained seropositive. Table1 [see Additional file 1] shows the seroprevalence by subgroup. The weighted prevalence [95% confidence interval] was 43.7% [35.9–51.6%] in the affected subdistricts of District A, which was not significantly different that of 29.7% [23.3–36.0%] in the affected subdistricts of District B. We detected no significant difference in the prevalence of neutralizing antibodies between the affected subdistricts and the non-affected subdistricts of both districts.

The prevalence among pregnant women in both districts was significantly lower than most of all other subgroups. The prevalence of pregnant participants (24.3% [20.1–28.8%] in district A, and 12.8% [9.7–16.5%] in district B) were not significant difference from non-pregnant participants aged 18–40 years in the same district (30.7% [20.5–42.4%] in district A and 14.4% [8.9–21.6%] in district B).

Table 2 [see Additional file 2] compares the prevalence of neutralizing antibodies among subgroups of non-pregnant participants. The prevalence were significantly associated with age and living near the natural water within 100 m. The prevalence of neutralizing antibodies was 20.3% among young adults aged 18–40 years, 35.4% among those aged 41–60 years and 46.5% among those aged more than 60 years.

Table 3 [see Additional file 3] shows the results of the multivariable logistic regression analysis. There was no significant effect of distance from the nearest index case’s house to the participant’s house. Only significant predictors for a subject having neutralizing antibodies included age more than 60 years.

**Fig. 2:**
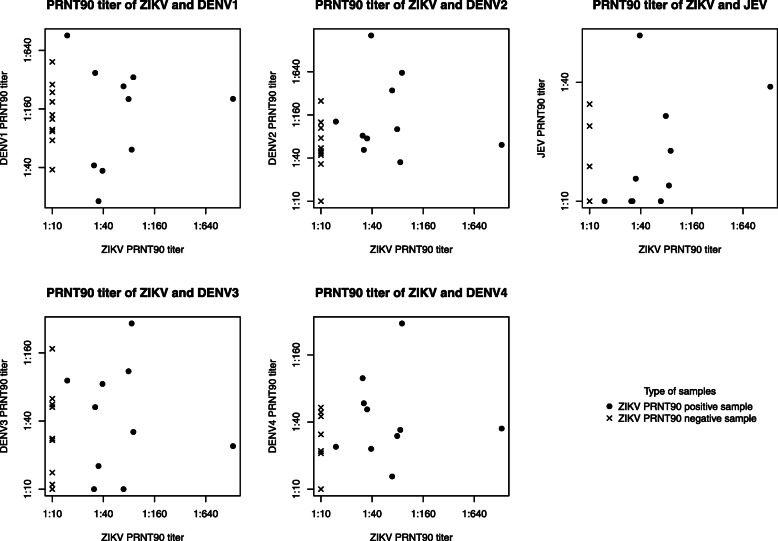
Relationship between PRNT90 titers of various flaviviruses in ZIKV seropositives and seronegatives

Figure 2 illustrates the cross-distribution of titers of neutralizing antibodies against various flaviviruses (Y-axes) and ZIKV (X-axis). The black dots scattered on the right represent positive ZIKV tests. The crosses on the left side at a titer of 1:10 represent samples that tested negative. Nearly all samples had positive tests against the other viruses, indicating that the majority of subjects with ZIKV negative tests had positive test results for neutralizing antibodies against other flaviviruses. Thus, a high proportion of ZIKV negative cases were harboring neutralizing antibodies against all study flaviviruses.

## Discussion

Approximately 18 months after the two ZIKV outbreaks in southern Thailand, one-third to nearly half of the population had neutralizing antibodies against the virus. The prevalence was not significantly different between outbreak and non-outbreak areas. Elderly groups were more likely to have this neutralizing antibody. Pregnant women had a significantly lower prevalence of neutralizing antibody than the non-pregnant group.

The prevalence of seropositivity reported in this study was in the range of that found in the post-outbreak area of French Polynesia (49% measured at 18 months post-outbreak) and Nicaragua (56% measured at 1 year post-outbreak), French Guiana (23.3% measured at 2 years post-outbreak) and Suriname (35.1% measured at 1 year post-outbreak) [28–31]. Our investigation was conducted 18 months after the outbreak when no active cases were detected. The immunity have developed in response to, or independent from, the ZIKV outbreak 18-month ago ZIKV, or is an artifact of the serological background of the ZIKV prior to the pandemic occurring. ZIKV was believed to be endemic in the Southeast Asia region for many years [12, 26, 32, 33].

Regardless of the nature of the source, more than half of the population are at risk of infection. The level of seroprevalence did not reach the theoretical threshold of herd immunity of 85.5%. This scenario is comparable with situation of dengue virus in Thailand. Even through the seroprevalence of DENV was as high as 79.2%, but there were around 100,000 cases reported annually [34]. In contrast with contagious diseases, spreading ability of arboviral diseases depends on vector and environmental factors.

Our results failed to demonstrate a dose-response relationship between seropositivity and distance to the nearest index case’s house. There was also no significant difference in seropositivity between outbreak areas and adjacent non-outbreak areas suggesting that the outbreak did not produce significant immunity in the population. This may be because the sizes of the ZIKV outbreak were very small compared to those in the Pacific Islands and the Americas where the numbers of cases exceeded 900,000 [10].

Our results, combined with those from a survey among healthy Thais in Central Thailand (seroprevalence of 70.4%[PRNT50 ≥ 10] and 20.2%[PRNT90 ≥ 20]), suggest that this Thai population were only partially protected by the antibody [26]. These levels of immunity may explain the low but sustained level of ZIKV transmission in Thailand as proposed by previous authors (Ruchusatsawat et al., 2019) [35].

Our results showed that older age is associated with seropositivity. This result contrasts with those of studies from Nicaragua, French Guiana and Suriname, where the Zika virus had been believed to be a de novo pathogen in the Americas during the outbreak [28–30]. The association between older age and seropositivity was also observed in a serosurvey in Thailand of other endemic flaviviruses such as dengue and chigunkunya [34, 36]. Therefore, this finding supports the theory that ZIKV has been circulating in the country for many year. The immunological cross-reactivity between Zika and other flaviviruses is well known [37, 38]. In this study, we used PRNT, which reflects whether or not a person is protected against a particular virus [25, 39]. Thus, we are concerned about cross-protection rather than cross-reaction. However, a high proportion of negative ZIKV PRNT cases with positive titers of other flaviviruses suggests that antibodies against those viruses may not completely protect individuals against ZIKV. Further studies in a greater cohort are needed to confirm the hypothesis that the endemic for other flaviviuses might not protect the population against ZIKV infection.

The low prevalence of neutralizing antibodies among pregnant women in these two outbreak areas is of important public health concern. Apart from age group, pregnancy can reduce the immunity of women making them more susceptible to many infections [40–44]. A low immunity against ZIKV in endemic areas would increase the risk of both the women and the fetuses to develop an infection, which can cause serious consequences, especially neurological deficit and microcephaly.

One main limitation of our study was that we examined the seroprevalence only 18 months after the outbreak. The initial and changing prevalence of neutralization in the population could, therefore, not be assessed. Our limited resources also allowed us to test neutralizing antibodies against other types of flavivirus in only a small number of subjects.

## Conclusions

The fact that more than half of the general population and more than three-quarters of the pregnant women were seronegative indicates a sustained risk for future ZIKV outbreaks. The community will therefore benefit from efficacious ZIKV vaccine once it becomes available.

## Supplementary Information

**Additional file 1: Table 1.** Seroprevalence of ZIKV neutralizing antibodies among subgroups of participants in District A and B

**Additional file 2: Table 2.** Univariable analysis exploring factors associated with ZIKV seropositivity in non-pregnant participants in the two sites combined. (a multi-page table)

**Additional file 3: Table 3.** Odds ratios for ZIKV seropositivity from the multivariate logistic regression analysis among non-pregnant participants in the two sites combined. (a multi-page table)

## Data Availability

The datasets used and/or analyzed during the current study are available from the corresponding author on reasonable request.

## Abbreviations

Adj OR: Adjusted odds ratio
CVD: Center for vaccine development
DENV: Dengue virus
JEV: Japanese encephalitis virus
OR: Odds ratio
PFU: Plaque-forming unit
PRNT: Plaque reduction neutralization test
RT-PCR: Reverse transcriptase-polymerase chain reaction
ZIKV: Zika virus
