# Supplementary material for: Survey on neutralizing antibodies against Zika virus eighteen months post-outbreak in two southern Thailand communities
Source: BMC Infect Dis. 2020 Dec 3;20:921. doi: 10.1186/s12879-020-05654-8 (PMC7711253; doi:10.1186/s12879-020-05654-8)
Supplement: Supplementary file 1 — Additional file 1: Table 1. Seroprevalence of ZIKV neutralizing antibodies among subgroups of participants in District A and B [file 12879_2020_5654_MOESM1_ESM.docx]

**Table 1** Seroprevalence of ZIKV neutralizing antibodies among subgroups of participants in District A and B

| **Participant subgroup** | **Total** | **PRNT90** | | | |
| --- | --- | --- | --- | --- | --- |
|  |  | **Seroprevalence** | | **Geometric mean titer** | |
|  |  | **(%** | **[95% CI])** |  |  |
| **District A** |  |  |  |  |  |
| Index cases | 10 | 50.0 | [18.7-81.3] | 29.5 | [16.0-54.4] |
| Affected subdistricts | 272 | 43.7† | [35.9-51.6] | 40.9 | [34.3-48.9] |
| Household member | 44 | 50.0 | [35.1-64.9] | 43.9 | [27.3-70.8] |
| Resident <100m | 36 | 44.4 | [28.0-60.9] | 41.7 | [24.7-70.3] |
| Resident 101-400m | 95 | 41.1 | [31.1-51.0] | 39.6 | [29.7-52.6] |
| Resident 401-1,000m | 97 | 44.3 | [34.4-54.3] | 40.4 | [29.3-55.7] |
| Non-affected subdistricts | 95 | 33.7 | [24.1-43.2] | 43.4 | [24.7-76.1] |
| Pregnant women | 400 | 24.3 | [20.1-28.8] | 55.4 | [43.4-70.6] |
| **District B** |  |  |  |  |  |
| Index cases | 8 | 50.0 | [15.7-84.3] | 50.7 | [8.4-306.3] |
| Affected subdistricts | 282 | 29.7† | [23.3-36.0] | 42.6 | [32.6-55.8] |
| Household member | 20 | 45.0 | [22.6-67.4] | 36.0 | [18.4-70.3] |
| Resident <100m | 73 | 24.7 | [14.7-34.6] | 26.1 | [19.6-34.9] |
| Resident 101-400m | 96 | 32.3 | [22.9-41.7] | 54.8 | [33.1-90.8] |
| Resident 401-1,000m | 93 | 20.4 | [12.2-28.7] | 48.6 | [25.7-91.9] |
| Non-affected subdistricts | 95 | 26.3 | [17.4-35.2] | 64.7 | [37.5-111.6] |
| Pregnant women | 405 | 12.8 | [9.7-16.5] | 39.6 | [28.8-54.5] |
