# Supplementary material for: Survey on neutralizing antibodies against Zika virus eighteen months post-outbreak in two southern Thailand communities
Source: BMC Infect Dis. 2020 Dec 3;20:921. doi: 10.1186/s12879-020-05654-8 (PMC7711253; doi:10.1186/s12879-020-05654-8)
Supplement: Supplementary file 2 — Additional file 2: Table 2. Univariable analysis exploring factors associated with ZIKV seropositivity in non-pregnant participants in the two sites combined. (a multi-page table) [file 12879_2020_5654_MOESM2_ESM.docx]

**Table 2** Univariable analysis exploring factors associated with ZIKV PRNT90 seropositivity in non-pregnant participants in the two sites combined

| **Variable** | |  | | **PRNT90 seropositivity** | | | | | | **P-value**  **(Rao-Scott)** |
| --- | --- | --- | --- | --- | --- | --- | --- | --- | --- | --- |
|  |  |  | | **District A** | | **District B** | | **Combined** | |  |
|  |  |  |  | **n** | **(%)** | **n** | **(%)** | **n** | **(%)** |  |
| Study site | |  |  |  |  |  |  |  |  | 0.079 |
|  | District A |  |  | 152 | (41.4) | - |  | 152 | (41.4) |  |
|  | District B |  |  | - |  | 102 | (27.1) | 102 | (27.1) |  |
| Family member with a history of ZIKV infection | | | |  |  |  |  |  |  | 0.141 |
|  | Yes |  |  | 22 | (50.0) | 9 | (45.0) | 31 | (48.4) |  |
|  | No |  |  | 130 | (40.2) | 93 | (26.1) | 223 | (32.8) |  |
| Age group (years) | |  |  |  |  |  |  |  |  | <0.001 |
|  | 18-40 |  |  | 23 | (30.7) | 19 | (14.4) | 42 | (20.3) |  |
|  | 41-60 |  |  | 74 | (44.0) | 46 | (26.9) | 120 | (35.4) |  |
|  | >60 |  |  | 55 | (44.4) | 37 | (50.0) | 92 | (46.5) |  |
| Gender | |  |  |  |  |  |  |  |  | 0.941 |
|  | Male |  |  | 52 | (36.1) | 54 | (34.4) | 106 | (35.2) |  |
|  | Female |  |  | 100 | (44.8) | 48 | (21.8) | 148 | (33.4) |  |
| Marital status | |  |  |  |  |  |  |  |  | 0.372 |
|  | Single |  |  | 13 | (27.1) | 4 | (12.9) | 17 | (21.5) |  |
|  | Married/cohabiting |  |  | 114 | (43.7) | 76 | (26.2) | 190 | (34.5) |  |
|  | Widowed/separated |  |  | 25 | (43.1) | 22 | (39.3) | 47 | (41.2) |  |
| Highest education level attained | |  |  |  |  |  |  |  |  | 0.305 |
|  | No formal education |  |  | 6 | (46.2) | 30 | (32.6) | 36 | (30.9) |  |
|  | Primary school |  |  | 83 | (45.4) | 60 | (31.1) | 143 | (38) |  |
|  | Secondary school or higher |  |  | 63 | (36.8) | 12 | (13.0) | 75 | (28.5) |  |
|  | |  |  | **PRNT90 seropositivity** | | | | | |  |
| **Variable** | |  |  | **District A** | | **District B** | | **Combined** | | **P-value** |
|  |  |  |  | **n** | **(%)** | **n** | **(%)** | **n** | **(%)** | **(Rao-Scott)** |
| Occupation | |  |  |  |  |  |  |  |  | 0.065 |
|  | Agricultural worker |  |  | 81 | (42.0) | 59 | (37.6) | 140 | (40.0) |  |
|  | Non-agricultural labourer |  |  | 11 | (30.6) | 13 | (21.7) | 24 | (25.0) |  |
|  | Private business owner |  |  | 18 | (43.9) | 7 | (16.3) | 25 | (29.8) |  |
|  | Unemployed |  |  | 36 | (46.2) | 22 | (20.0) | 58 | (39.8) |  |
|  | Others |  |  | 6 | (31.6) | 1 | (14.3) | 7 | (26.9) |  |
| History of self-reported dengue infection | | | |  |  |  |  |  |  | 0.632 |
|  | Yes |  |  | 14 | (31.1) | 8 | (24.2) | 22 | (28.2) |  |
|  | No |  |  | 138 | (42.9) | 94 | (27.3) | 232 | (34.8) |  |
| History of self-reported chikungunya infection | | | |  |  |  |  |  |  | 0.256 |
|  | Yes |  |  | 22 | (43.1) | 39 | (31.7) | 61 | (35.5) |  |
|  | No |  |  | 130 | (41.1) | 63 | (24.8) | 193 | (33.9) |  |
| Frequency of personal repellent use | | | |  |  |  |  |  |  | 0.135 |
|  | Regularly (> once per week) |  |  | 16 | (47.1) | 10 | (35.7) | 26 | (41.9) |  |
|  | Irregularly |  |  | 136 | (40.8) | 92 | (26.4) | 228 | (33.4) |  |
| Frequency of repellent use in household | | | |  |  |  |  |  |  | 0.826 |
|  | Regularly (> once per week) |  |  | 29 | (37.2) | 36 | (39.1) | 65 | (38.2) |  |
|  | Irregularly |  |  | 123 | (42.6) | 66 | (23.2) | 189 | (32.9) |  |
| Frequency of cleaning around household | | | |  |  |  |  |  |  | 0.375 |
|  | Regularly (> once per week) |  |  | 56 | (37.8) | 50 | (25.5) | 106 | (30.8) |  |
|  | Irregularly |  |  | 96 | (44.0) | 52 | (28.7) | 148 | (37.1) |  |
|  |  |  |  |  |  |  |  |  |  |  |
|  |  |  |  |  |  |  |  |  |  |  |
|  | |  |  | **PRNT90 seropositivity** | | | | | |  |
| **Variable** | |  |  | **District A** | | **District B** | | **Combined** | | **P-value** |
|  |  |  |  | **n** | **(%)** | **n** | **(%)** | **n** | **(%)** | **(Rao-Scott)** |
| Frequency of larvicide use | | | |  |  |  |  |  |  | 0.475 |
|  | Regularly (> once per month) |  |  | 81 | (44.3) | 12 | (27.9) | 93 | (41.2) |  |
|  | Irregularly |  |  | 71 | (38.6) | 90 | (26.9) | 161 | (31.1) |  |
| Sealing of all household entrances | | | |  |  |  |  |  |  | 0.071 |
|  | Yes |  |  | 9 | (50.0) | 0 | (0.0) | 9 | (50.0) |  |
|  | No |  |  | 143 | (41.0) | 102 | (27.1) | 245 | (33.7) |  |
| Use of mosquito net | | | |  |  |  |  |  |  | 0.828 |
|  | Yes |  |  | 75 | (41.9) | 81 | (25.6) | 156 | (31.5) |  |
|  | No |  |  | 77 | (41.0) | 21 | (34.4) | 98 | (39.4) |  |
| Other household within 100m | | | |  |  |  |  |  |  | 0.308 |
|  | Yes |  |  | 126 | (41.6) | 93 | (26.6) | 219 | (33.5) |  |
|  | No |  |  | 26 | (40.6) | 9 | (33.3) | 35 | (38.5) |  |
| Plantation within 100m of household | | | |  |  |  |  |  |  | 0.275 |
|  | Yes |  |  | 122 | (40.3) | 80 | (26.8) | 202 | (33.6) |  |
|  | No |  |  | 30 | (46.9) | 22 | (27.8) | 52 | (36.4) |  |
| Natural water within 100m of household | | | |  |  |  |  |  |  | 0.018 |
|  | Yes |  |  | 21 | (41.3) | 19 | (27.1) | 40 | (33.3) |  |
|  | No |  |  | 131 | (42.0) | 83 | (27.0) | 214 | (34.3) |  |
| Uncovered water container(s) within a 100m radius | | | |  |  |  |  |  |  | 0.095 |
|  | Yes |  |  | 55 | (41.7) | 12 | (32.4) | 67 | (39.6) |  |
|  | No |  |  | 97 | (41.3) | 90 | (26.5) | 187 | (32.5) |  |
