# Supplementary material for: Survey on neutralizing antibodies against Zika virus eighteen months post-outbreak in two southern Thailand communities
Source: BMC Infect Dis. 2020 Dec 3;20:921. doi: 10.1186/s12879-020-05654-8 (PMC7711253; doi:10.1186/s12879-020-05654-8)
Supplement: Supplementary file 3 — Additional file 3: Table 3. Odds ratios for ZIKV seropositivity from the multivariate logistic regression analysis among non-pregnant participants in the two sites combined. (a multi-page table) [file 12879_2020_5654_MOESM3_ESM.docx]

**Table 3** Odds ratios for ZIKV PRNT90 seropositivity from the multivariate logistic regression analysis among non-pregnant participants in the two sites combined

| **Variables** | | **Crude OR** | **(95% CI)** | **Adj OR** | **(95% CI)** | **P-value (Wald's test)** | **P-value (LR test)** |
| --- | --- | --- | --- | --- | --- | --- | --- |
| Study site | |  |  |  |  |  | 0.711 |
|  | District A | Ref |  |  |  |  |  |
|  | District B | 0.65 | (0.40-1.05) | 0.90 | (0.50-1.60) | 0.711 |  |
| Distance to the nearest index case's house | | |  |  |  |  | 0.279 |
|  | 0 m (household contact) | Ref |  |  |  |  |  |
|  | 1 – 100 m | 0.45 | (0.20-1.04) | 0.37 | (0.13-1.11) | 0.078 |  |
|  | 101 – 400 m | 0.62 | (0.28-1.35) | 0.58 | (0.21-1.64) | 0.307 |  |
|  | 401 – 1,000 m | 0.81 | (0.37-1.79) | 0.52 | (0.18-1.55) | 0.244 |  |
|  | >1,000 m (other subdistrict) | 0.50 | (0.23-1.08) | 0.41 | (0.14-1.16) | 0.095 |  |
| Age group (years) | |  |  |  |  |  | 0.003 |
|  | 18-40 | Ref |  |  |  |  |  |
|  | 41-60 | 2.51 | (1.29-4.91) | 1.94 | (0.93-4.08) | 0.079 |  |
|  | >60 | 4.91 | (2.42-9.97) | 3.86 | (1.72-8.68) | 0.001 |  |
| Occupation | |  |  |  |  |  | 0.279 |
|  | Unemployed | Ref |  |  |  |  |  |
|  | Agricultural worker | 1.26 | (0.72-2.21) | 1.26 | (0.69-2.31) | 0.451 |  |
|  | Non-agricultural laborer | 0.32 | (0.13-2.21) | 0.46 | (0.18-1.19) | 0.111 |  |
|  | Private business owner | 0.98 | (0.09-2.37) | 1.43 | (0.59-3.44) | 0.426 |  |
|  | Others | 0.46 | (0.43-2.21) | 0.72 | (0.14-3.71) | 0.698 |  |
| Frequency of personal repellent use | |  |  |  |  |  | 0.163 |
|  | Irregular | Ref |  |  |  |  |  |
|  | Regularly (> once per week) | 1.97 | (0.80-4.87) | 2.04 | (0.75-5.55) | 0.163 |  |
|  | | | |  |  |  |  |
|  | | | |  |  |  |  |
| All household entrances sealed | | | |  |  |  | 0.256 |
|  | No | Ref |  |  |  |  |  |
|  | Yes | 3.50 | (0.83-14.8) | 2.42 | (0.53-11.1) | 0.256 |  |
| Natural water within 100m of household | | | | |  |  | 0.067 |
|  | No | Ref |  |  |  |  |  |
|  | Yes | 0.48 | (0.25-0.89) | 0.54 | (0.28-1.04) | 0.067 |  |
| Uncovered water container(s) within a 100m radius | | | |  |  |  | 0.633 |
|  | No | Ref |  |  |  |  |  |
|  | Yes | 1.61 | (0.92-2.84) | 1.17 | (0.62-2.20) | 0.633 |  |
